# Functional implications of Rab27 GTPases in Cancer

**DOI:** 10.1186/s12964-018-0255-9

**Published:** 2018-08-06

**Authors:** Zhihong Li, Rui Fang, Jia Fang, Shasha He, Tang Liu

**Affiliations:** 1 Department of Orthopaedics, The Second Xiangya Hospital, Central South University, Changsha, Hunan China; 2 Department of Neurology, The Second Xiangya Hospital, Central South University, Changsha, Hunan China; 3 Department of Oncology, The Second Xiangya Hospital, Central South University, Changsha, Hunan People’s Republic of China

**Keywords:** Rab27, Small GTPase, Exosome, Tumor microenvironment, Cancer

## Abstract

**Background:**

The Rab27 family of small GTPases promotes the progression of breast cancer, melanoma, and other human cancers. In this review, we discuss the role of Rab27 GTPases in cancer progression and the potential applications of these targets in cancer treatment.

**Main body:**

Elevated expression of Rab27 GTPases is associated with poor prognosis and cancer metastasis. Moreover, these GTPases govern a variety of oncogenic functions, including cell proliferation, cell motility, and chemosensitivity. In addition, small GTPases promote tumor growth and metastasis by enhancing exosome secretion, which alters intracellular microRNA levels, signaling molecule expression, and the tumor microenvironment.

**Conclusion:**

Rab27 GTPases may have applications as prognostic markers and therapeutic targets in cancer treatment.

## Background

Rabs are small GTPases (20-25 kDa) that belong to the Rab protein family, which is composed of more than 70 mammalian members and forms the largest branch of the Ras superfamily [1]. Rabs are important regulators of vesicle trafficking, which is one of the mechanisms controlling cellular functions, including cell proliferation / invasion, signal transduction, and protein transport [2, 3]. Rabs are involved in cancer development and progression. For example, Rab25 acts as an oncogene in renal, ovarian, and breast cancers [4] and Rab27 is a major regulator of exosome secretion and possesses oncogenic function.

Rab27 is a Rab protein that is widely conserved in metazoans but is not found in yeasts or plants [5]. In vertebrates, Rab27 consists of two isoforms, Rab27A and Rab27B, which are encoded by two different genes [6]. Rab27A is widely expressed in melanocytes, cytotoxic T lymphocytes, and various secretory cells, including exocrine, endocrine, ovarian, and hematopoietic cells [7, 8]. Dysfunction of Rab27A has been reported to cause a human hereditary diseases, type 2 Griscelli syndrome [9], and is related to chronic inflammation [10]. Rab27B is mainly expressed in platelets and in the stomach, large intestine, pancreas, pituitary, and bladder [11–13]. To date, no disease-causing mutation of Rab27B has been identified [8].

Rab27A and Rab27B are important molecules regulating vesicle trafficking. They control vesicle transport by acting as molecular switches that oscillate between the GTP-bound active form and GDP-bound inactive form. The status of Rab27, as well as other Rabs, is controlled by two key regulatory enzymes, guanine nucleotide exchanged factor (activator) and GTPase-activating protein (inactivator) [14, 15]. In the active form, Rab27 recruits effector proteins and coordinates the vesicle trafficking process, which is involved in vesicle sorting, uncoating, motility, tethering, and fusion [16, 17]. Eleven Rab27-specific effectors have been identified. Based on the organization of their domains, the eleven Rab27 effectors are classified into three groups, i.e., the synaptotagmin-like protein (Slp), the Slp homologue lacking C2 domains protein (Slac2), and mammalian uncoordinated 13–4 (Munc13–4). The spatiotemporal recruitment of these effectors is crucial for determining the efficiency and specificity of Rab27-mediated exocytosis [18].

Each Rab GTPase localizes in distinct subcellular organelles and governs specific vesicles transport pathways between different cellular compartments [2, 3]. Rab27A and Rab27B have been reported to be associated with and regulate the transport of lysosome-related organelles, such as melanosomes in melanocytes, lytic granules in cytotoxic T lymphocytes, and dense granules in platelets [19–21]. Recent studies have revealed that Rab27A and Rab27B control exosome secretion in various cell types, including dendritic cells [22], cervical cancer cells [23], breast cancer cells [24, 25], melanoma cells [26], bladder cancer cells [27], and lung cancer cells [28].

Although Rab27A and Rab27B share high sequence similarity (71% amino acid sequence identity) [29] and recruit the same effector proteins [30], the two Rab27 isoforms have been shown to function differently, even in the same cell type [31]. In a study by Ostrowski et al., both Rab27A and Rab27B were found to promote exosome secretion in HeLa cells [23]. However, they were shown to have different roles in the exosomal pathway; Rab27A regulates docking and membrane fusion of multivesicular endosomes (MVEs), whereas Rab27B participates in the transfer of membranes from the trans-Golgi network (TGN) to MVEs [23].

Because the biological role of Rab27A/B is crucial for maintaining proper cellular function, aberrant expression of Rab27A/B may lead to cancer development. Elevated expression of Rab27 was found in a hybrid Xiphophorus melanoma model, in which melanoma is inducible by ultraviolet light (UVB) exposure. Moreover, the expression of Rab27 was further upregulated upon UVB exposure, suggesting that Rab27 may play a role in melanomagenesis [32]. Altered expression of Rab27A/B is observed in various human cancers and contributes to cancer progression.

In this review, we discuss the biological functions of Rab27 and the role of Rab27 in cancer.

### Oncogenic function of Rab27

Rab27 has been shown to play a crucial role in cancer progression. Both Rab27A and Rab27B have been reported to promote cell proliferation, enhance cell invasion, and increase chemoresistance of cancer [33] (Table 1). The oncogenic function of Rab27A/B is likely due to its function in regulating exosome secretion, which modulates cancer cell function and the tumor microenvironment.

**Table 1:** Biological function of Rab27A and Rab27B

	Rab27A	Rab27B
Promote cell growth (in vitro)	• Melanoma cells (WM1385, WM1960) [34]	• Breast cancer cells (MCF-7, T47D, ZR75.1) [35]
Promote tumor growth (in vivo)	• Melanoma cells (SK-Mel-28, B16-F10) [26] • Breast cancer cells (4T1) [25]	• Breast cancer cells (MCF-7) [35]
Promote cell invasion (in vitro)	• Bladder cancer cells (T24, FL3) [27]	• Bladder cancer cells (T24, FL3) [27] • Breast cancer cells (MCF-7, T47D) [35]
Promote tumor metastasis (in vivo)	• Melanoma cells (SK-Mel-28, B16-F10) [26] • Breast cancer cells (4T1) [35]	• Breast cancer cells (MCF-7) [35]
Increase chemoresistance (in vitro)	––	• Breast cancer cells (MCF-7) [37]
Increase exosome secretion (in vitro)	• Bladder cancer cells (T24, FL3) [27] • Cervical cancer cells (HeLa) [23] • Breast cancer cells (MDA-MB-231, 4T1) [24, 25] • Melanoma cells (SK-Mel-28, B16-F10) [26] • Lung adenocarcinoma cells (A529) [28]	• Bladder cancer cells (T24, FL3) [27] • Cervical cancer cells (HeLa) [23] • Increase V-ATPase in exosome secretion of breast cancer cells (MCF-7) [49] • Increase HSP90α in exosome secretion of breast cancer cells (MCF-7) [35]

#### Cell growth and tumor development

Both Rab27A and Rab27B enhance cell proliferation and tumor development. Rab27A knockdown by shRNA transfection suppresses in vitro cell growth of melanoma cells (WM1385 and WM1960) [34], and suppresses in vivo tumor growth of xenografts derived from human melanoma cells (SK-Mel-28) and murine melanoma cells (B16-F10) [26]. The function of Rab27A in regulating tumor growth was further supported by the results of a study by Bobrie et al., in which Rab27A blockade was found to reduce primary tumor growth of xenografts derived from 4 T1 murine mammary carcinoma cells [25]. The function of Rab27B in regulating tumor growth was supported by a study by Hendrix et al., in which knockdown of Rab27B by siRNA transfection was found to reduce in vitro cell proliferation of MCF-7 breast cancer cells [35]. Conversely, ectopic overexpression of Rab27B enhances the proliferation of MCF-7, T47D, and ZR75.1 breast cancer cells in the presence of low serum concentrations [35]. Overexpression of Rab27B promotes in vivo tumor growth and the development of hemorrhagic ascites in the peritoneal cavity of animals with MCF-7 breast cancer cell-derived xenografts [35].

#### Cell invasion and tumor metastasis

Recent reports have indicated the involvement of Rab27A and Rab27B in promoting cell invasion and tumor metastasis. Knockdown of Rab27A suppresses in vitro invasion of bladder cancer cells (T24, FL3) [27]. Moreover, Rab27A knockdown reduces lung metastasis of tumors derived from human melanoma cells (SK-Mel-28) [26], murine melanoma cells (B16-F10) [26], and murine 4 T1 mammary carcinoma cells [25]. Knockdown of Rab27B by siRNA suppresses in vitro cell invasion of bladder cancer cells (T24, FL3) [27]. Additionally, ectopic overexpression of Rab27B enhances in vitro cell invasion of breast cancer cells (MCF-7, T47D) [35].Overexpression of Rab27B also increases in vivo muscular invasion of xenografts derived from MCF-7 breast cancer cells [35].

Immunohistochemical analysis of Rab27B, mesenchymal markers, and epithelial markers was performed in 221 tumor specimens from patients with invasive breast cancer. Spearman’s correlation analysis showed that Rab27B expression was positively correlated with the expression levels of mesenchymal markers (Vimentin and Fibronectin) and inversely correlated with epithelial markers (E-cadherin and β-catenin). Thus, overexpression of Rab27B contributed to the EMT process in breast cancer progression [36].

#### Chemoresistance

The involvement of Rab27 in regulating the chemosensitivity of cancer cells has not been studied extensively. Ectopic overexpression of Rab27B induces doxorubicin resistance in breast cancer cells [37] and MCF-7 cells stably expressing Rab27B are significantly more resistant to doxorubicin (IC50 = 1.56 μM), compared with that in control MCF-7 cells (IC50 = 0.47 μM) [37]. Cell overexpressing Rab27B were found to have less doxorubicin-induced poly (ADP-ribose) polymerase (PARP) cleavage, indicating that Rab27B suppresses PARP-dependent cell death pathways, leading to increased doxorubicin resistance [37]. Moreover, Rab27A transfection induces cisplatin resistance in BIU-87 human bladder cancer cells, and Rab27A depletion by siRNA reduces cisplatin resistance in 5637 human bladder cancer cells. The nuclear factor (NF)-κB inhibitor BAY 11–7082 abolishes the effects of Rab27A on cisplatin resistance, suggesting that the NF-κB signaling pathway has a role in mediating the effects of Rab27A on cisplatin resistance [38].

### Mechanisms of Rab27 functions

#### Up-stream regulators of Rab27

Altered expression of Rab27A/B is commonly observed in cancer. However, the mechanisms leading to alterations in Rab27A/B expression are not fully understood. Recently, Rab27A expression was reported to be regulated by microRNA and gene copy number amplifications.

The expression levels of several Rabs are controlled by microRNAs. For example, downregulation of miR-9 elevates the expression of Rab34 in gastric cancer [39], whereas downregulation of let-7d increases the expression of Rab25 in renal cell carcinoma [40]. Rab27A is a direct target of miR-124a, which decreases the level of Rab27A in insulin-secreting cells [41]. However, whether the altered expression of Rab27A/B in cancer is due to aberrant microRNAs expression is still unclear.

Copy number amplification is another cause of Rab27 upregulation. Rab27A is encoded by the Rab27A gene located at chromosome 15q21.3. Rab27A gene amplification was found in 101 melanoma tumor samples [34], indicating that elevated Rab27A mRNA expression may be due to copy number amplification in melanoma [34].

#### Down-stream effectors of Rab27

##### Exosome secretion in regulation of cancer cell function

Rab27A and Rab27B promote exosome secretion in various types of cancer. Knockdown of Rab27A or Rab27B reduces exosome release in T24 and FL3 bladder cancer cells and HeLa cervical cancer cells [23, 27]. Moreover, Rab27A knockdown suppresses exosome secretion in breast cancer cells (MDA-MB-231 and 4 T1) [24, 25], melanoma cells (SK-Mel-28 and B16-F10) [26], and lung adenocarcinoma cells (A529) [28].

Exosomes are nano-sized membrane vesicles with a diameter of 40–100 nm [42]. These organelles were first described as vesicles released from multivesicular endosomes (MVEs) in reticulocytes for the removal of obsolete transferring receptors (TFR) [43]. Upon MVE fusion with the plasma membrane, intraluminal vesicles are released into the extracellular space as exosomes, a process regulated by Rab27A/B as well as other exosome-regulating molecules [23, 44, 45]. Exosomes contain various types of proteins [46], mRNAs, and microRNAs [47]. Secretion of exosomes regulates the oncogenic function of cancer cells by reducing the intracellular content of microRNAs and altering the distribution of plasma membrane proteins. Moreover, transfer of functional proteins / microRNAs by exosomal secretion promotes oncogenic signaling in recipient cells upon delivery of cargo molecules [48]. Clinically, higher levels of protein-rich exosomes are associated with poor prognosis in patients with melanoma [26].

**Fig. 1:**
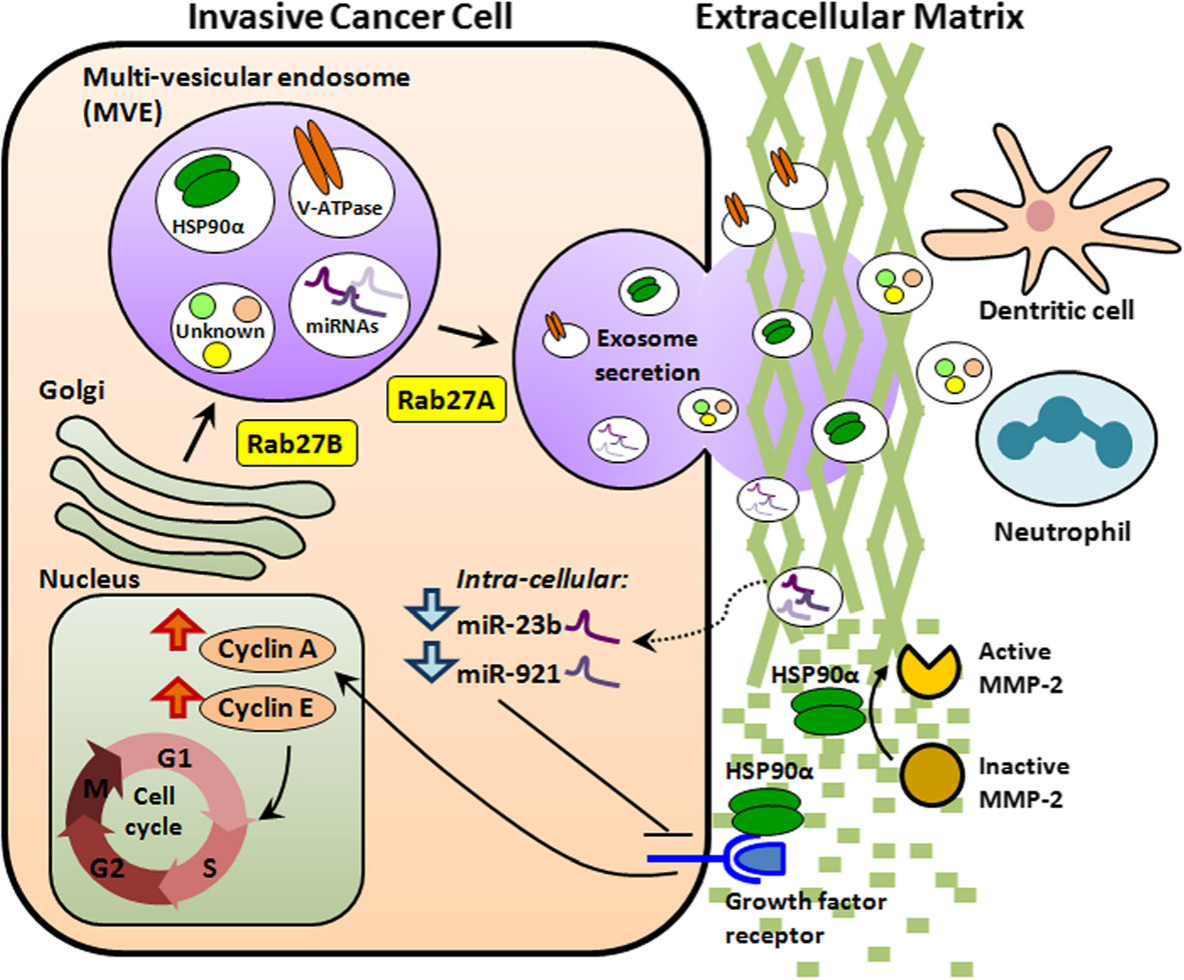
Schematic diagram of the mechanisms through which Rab27 GTPases regulate invasive cancer growth. Rab27A and Rab27B enhance invasive cancer growth by promoting exosome secretion. Elevated exosome secretion leads to 1) reduced intracellular tumor-suppressive microRNAs, including miR-23b and miR-921; 2) increased cellular signaling molecules, including VATPase and HSP90α, which regulate the cell cycle, enhance the G1 to S-phase transition, stimulate cell growth, and induce focal activation of MMP-2, thereby increasing extracellular matrix degradation and facilitating cell invasion; and 3) increased secretion of unknown substances that cause mobilization of neutrophils and dendritic cells, thereby modulating the tumor microenvironment and enhancing cancer cell metastasis

Rab27A and Rab27B promote oncogenic functions predominantly by increasing exosome secretion. Elevated exosome secretion modulates the tumor microenvironment and alters intracellular levels of microRNAs and cellular signaling molecules (Fig. 1). Rab27A/B-induced exosome secretion reduces the intracellular level of tumor-suppressive microRNAs, including miR-23b and miR-921. Decreased levels of miR-23b and miR-921 promote cancer growth and metastasis [27]. In a study of bladder cancer, knockdown of Rab27A or Rab27B attenuated cell invasion, reduced exosome release, and increased intracellular levels of miR-23b and miR-921. The increase in intracellular miR-23b and miR-921 levels after Rab27A/B knockdown was thought to be caused by reduction of exosome release. Moreover, miR-23b knockdown reverses the attenuation of cell invasion due to Rab27B knockdown. These findings indicated that Rab27A/B promoted cancer metastasis by enhancing exosome release, which increases the removal of tumor-suppressive microRNAs from cancer cells [27].

Exosomes derived from Rab27A/B-overexpressing breast cancer cells contain abundant signaling molecules that promote cancer progression. Additionally, the exosomes derived from MCF-7 cells stably expressing Rab27B have high amounts of V-ATPase [49]. Knockdown of V-ATPase reverses the cell growth stimulatory effects of Rab27B overexpression. V-ATPase knockdown reduces the expression of cyclin A / cyclin E and blocks the G1 to S-phase transition. Thus, elevation of V-ATPase levels, due to Rab27B-dependent exosome secretion, stimulates cell growth through cell cycle regulation [49]. In another study of breast cancer, the heat-shock protein HSP90α was found in the exosomes isolated from Rab27B-overexpressing cells [35]. HSP90α secretion was found to be 7-fold higher in conditioned medium obtained from Rab27B overexpressing cells than in that obtained from control cells [35]. Anti-HSP90α antibodies reverse the increase in cell proliferation rate and cyclin A expression in Rab27B-overexpressing cells, suggesting that Rab27B promotes cancer growth by inducing exosomal secretion of HSP90α. Moreover, HSP90α has been shown to increase the activation of matrix metalloproteinase 2 (MMP-2), a protease that degrades the extracellular matrix [50]. Increased HSP90α-induced MMP-2 activation may mediate the effects of Rab27B on promoting cancer metastasis.

#### Exosome secretion regulates the tumor microenvironment

Rab27A/B-dependent exosome secretion leads to the formation of a supportive tumor environment that promotes cancer growth. Cancer progression is not only driven by transformed epithelial cells but also the tumor-promoting stromal environment [51]. Cancer cells and stromal cells, including fibroblasts, mesenchymal cells, bone marrow-derived endothelial cells, immune cells, and adipocytes, interact with each other to form tumor ecosystems [52–54]. Exosome secretion from both cancer cells and stromal cells facilitates the communication between different cell types in tumor ecosystems, which provide a metastatic niche that support tumor growth and metastasis [55].

Rab27A-dependent exosome release has been shown to promote tumor growth and metastasis by increasing the mobilization of neutrophils [25]. Neutrophils are immune cells of the innate immune system and provide the first line of defense against pathogens [56]. A high neutrophil-to-lymphocyte ratio (NLR) is associated with adverse overall survival and disease-free survival in patients with breast cancer [57], likely due to induction of inflammation and suppression of lymphocyte activities [58, 59]. In a study by Bobrie et al., mice with tumors derived from Rab27A-knockdown murine 4 T1 breast cancer cells were found to have less exosome secretion, less systemic accumulation of neutrophils, and reduced tumor growth / lung metastasis [25]. Exosomes collected from Rab27A-expressing cells promote tumor growth of Rab27A-knockdown cells and increase systemic accumulation of neutrophils, suggesting that Rab27A-dependent exosome secretion enhances the mobilization of neutrophils, thereby promoting tumor growth and metastasis [25].

According to a study by Peinado et al., Rab27A-dependent exosome release promotes education and mobilization of bone marrow-derived cells (BMDCs), supporting tumor growth and metastasis [26]. Rab27A knockdown in murine B16-F10 melanoma cells decreases exosome secretion, prevents bone marrow education, and reduces tumor growth and metastasis. Although the mechanisms are not fully understood, Rab27A-dependent exosome secretion has been shown to contribute to the mobilization of BMDCs, which leads to increased tumor growth and metastasis [26].

In addition to regulating exosome secretion in cancer cells, Rab27A/B also regulate exosome secretion in stromal cells. Rab27A/B double-knockout bone marrow-derived dendritic cells (BMDDCs) have both decreased exosome secretion and reduced miR-155 and miR-146a levels in exosomes [22]. MiR-155 and miR-146a are critical microRNAs that regulate inflammation. These miRNAs are released from dendritic cells by exosomal secretion and subsequently taken up by other recipient dendritic cells. Following uptake, these microRNAs regulate the expression of inflammatory genes [22]. Inflammation is an important factor enhancing the formation of the cancer-promoting microenvironment. Further studies are needed to identify the roles of Rab27 in regulating the cancer microenvironment by enhancing exosomal release of microRNAs from stromal cells.

Rab27A and Rab27B influence neutrophil recruitment by regulating vesicle trafficking of neutrophils. Rab27A regulates exocytosis of tertiary and specific granules in human neutrophils [60], whereas Rab27B induces granule exocytosis in neutrophils and promotes neutrophil migration [31]. Rab27A/B double-knockout (Rab27DKO) neutrophils exhibit impaired transwell migration in vitro in response to pro-inflammatory factors, macrophage inflammatory protein 2 (MIP-2), and leukotriene B4 (LTB4) [61]. Further studies are needed to determine whether Rab27A/B-induced neutrophil recruitment is involved in enhancing the formation of the tumor-promoting microenvironment.

### Clinical implications of Rab27 in cancer

The majority of clinicopathological findings indicate that Rab27A and Rab27B have oncogenic roles in cancer (Table 2). Elevated expression of Rab27A has been demonstrated in tumor specimens from patients with melanoma [34] and hepatocellular carcinoma [62], whereas elevated expression of Rab27B has been found in tumor specimens from patients with breast cancer [35, 36]. High expression of Rab27B is associated with reduced survival times in patients with breast cancer [27, 36] and bladder cancer [27]. In a study of hepatocellular carcinoma, high tumor expression of either Rab27A or Rab27B was shown to be associated with low patient survival rates, whereas high tumor expression of both Rab27A and Rab27B was associated with poor survival [62].

**Table 2:** Clinical implication of Rab27A and Rab27B in cancer

	Rab27A	Rab27B
Elevated Expression	• Melanoma [34] • Hepatocellular carcinoma [62]	• Breast cancer [35, 36] • Bladder cancer [27]
High expression correlated with poor prognosis	• Hepatocellular carcinoma [62]	• Breast cancer [36] • Hepatocellular carcinoma [62]
High expression correlated with lymph-node metastasis	• Hepatocellular carcinoma [62]	• Breast cancer [35, 36] • Hepatocellular carcinoma [62]

High expression of Rab27A or Rab27B is significantly correlated with advanced TNM classification in hepatocellular carcinoma [62]. Moreover, in a study of two independent cohorts of patients with estrogen receptor-positive breast cancer, high expression of Rab27B was shown to be correlated with lymph-node metastasis and pathological grade [35, 36], suggesting a role of Rab27B in promoting cancer metastasis.

Although Rab27 has been shown to be predominantly oncogenic, this protein has also been reported to act as a tumor suppressor in colorectal cancer and prostate cancer. Rab27A is downregulated in colorectal cancer [63], and reduced expression of Rab27A in colorectal cancer is associated with poor patient survival rates, advanced TNM stage, distant metastasis, and local recurrence [63]. Rab27A and Rab27B are frequently downregulated in advanced prostate cancer and are inversely correlated with prostate cancer outcomes. The expression levels of both proteins are dependent on vacuolar protein-sorting-associated protein 36 [64].

The role of Rab27 in bladder cancer is controversial. Ostenfeld et al. conducted a thorough study and showed that Rab27B acts as an oncogene. Elevated levels of Rab27B were found to be associated with poor prognosis in two independent cohorts of patients with bladder cancer corresponding to 196 and 294 tumors [27]. The cancer-promoting role of Rab27 was confirmed in vitro; knockdown of Rab27A or Rab27B suppressed cell invasion in bladder cancer cells [27]. However, in another study conducted by Ho et al., the expression levels of Rab27A and Rab27B were shown to be downregulated in two independent bladder cancer data sets corresponding to 152 and 75 tumors [65]. Further studies are needed in order to confirm the roles of Rab27 GTPases in bladder cancer.

The majority of clinicopathological evidence has indicated that elevated Rab27A/B expression is associated with shorter survival times, more advance cancer stages, and distant metastasis of cancer. Thus, Rab27A/B may be prognostic markers and therapeutic targets. However, more studies are needed to confirm the roles of Rab27A/B in cancer because Rab27A and Rab27B have been reported to have cancer type-dependent functions; the roles of Rab27A/B in cancer remain elusive.

## Conclusion and perspectives

Rab27A and Rab27B act as oncogenes in cancer. Biological and clinicopathological findings have revealed that elevated Rab27A/B expression levels affect cancer progression and survival in patients. In addition to cancer biomarkers, such as microRNAs and E-cadherin [66–70], Rab27A and Rab27B are also potential prognostic markers and therapeutic targets. The development of Rab27A/B-specific inhibitors, which are currently not available, is needed to investigate the possible use of Rab27A/B-targeting agents to improve cancer therapy. The mechanisms that lead to elevated expression of Rab27A/B in cancer are still unclear. To date, only one report has demonstrated Rab27A gene amplification in melanoma [34]. Further studies are needed to identify the mechanisms that lead to Rab27A/B overexpression in order to develop novel methods for cancer prevention. Rab27A and Rab27B promote cancer progression by increasing exosome secretion, which enhances the formation of a tumor-supporting microenvironment. Improving our understanding of the mechanisms through which Rab27A and Rab27B regulate exosome secretion will facilitate the identification of possible therapeutic interventions to improve cancer treatment.

## Abbreviations

BMDCs: Bone marrow–derived cells
BMDDCs: Bone marrow-derived dendritic cells
LTB4: Leukotriene B4
MIP-2: Macrophage inflammatory protein 2
MMP-2: Matrix metalloproteinase 2
Munc13–4: Mammalian uncoordinated 13–4
MVEs: Multivesicular endosomes
NF: Nuclear factor
NLR: Neutrophil-to-lymphocyte ratio
PARP: Poly (ADP-ribose) polymerase
Rab27DKO: Rab27A/B double-knockout
Slac2: Slp homologue lacking C2 domains protein
Slp: Synaptotagmin-like protein
TFR: Transferring receptors
TGN: Trans-Golgi network
UVB: Ultraviolet light
